# Interactive residual coordinate attention and contrastive learning for infrared and visible image fusion in triple frequency bands

**DOI:** 10.1038/s41598-023-51045-9

**Published:** 2024-01-02

**Authors:** Zhihua Xie, Sha Zong, Qiang Li, Peiqi Cai, Yaxiong Zhan, Guodong Liu

**Affiliations:** 1 Key Lab of Optic-Electronic and Communication, Jiangxi Science and Technology Normal University, Nanchang, China; 2 Nanchang Key Laboratory of Failure Perception & Green Energy Materials Intelligent Manufacturing, Nanchang, China; 3 Zhejiang University of Illinois at Urbana-Champaign Institute, Zhejiang University, Haining, China; 4 Jiangxi Coinfa Technology Co. Ltd, Nanchang, China

**Keywords:** Computer science, Information technology

## Abstract

The auto-encoder (AE) based image fusion models have achieved encouraging performance on infrared and visible image fusion. However, the meaningful information loss in the encoding stage and simple unlearnable fusion strategy are two significant challenges for such models. To address these issues, this paper proposes an infrared and visible image fusion model based on interactive residual attention fusion strategy and contrastive learning in the frequency domain. Firstly, the source image is transformed into three sub-bands of the high-frequency, low-frequency, and mid-frequency for powerful multiscale representation from the prospective of the frequency spectrum analysis. To further cope with the limitations of the straightforward fusion strategy, a learnable coordinate attention module in the fusion layer is incorporated to adaptively fuse representative information based on the characteristics of the corresponding feature maps. Moreover, the contrastive learning is leveraged to train the multiscale decomposition network for enhancing the complementarity of information at different frequency spectra. Finally, the detail-preserving loss, feature enhancing loss and contrastive loss are incorporated to jointly train the entire fusion model for good detail maintainability. Qualitative and quantitative comparisons demonstrate the feasibility and validity of our model, which can consistently generate fusion images containing both highlight targets and legible details, outperforming the state-of-the-art fusion methods.

## Introduction

The image fusion technique, as an important branch of information fusion, belongs to the quality enhancement in the image processing discipline. Theoretically, image fusion attempts to integrate complementary information from multiple modality images, which are captured from the same scene by different sensors. Owing to this endeavor, the fusion image will contain diverse meaningful information from multiple original images for better visual effects^1^. To be specific, infrared imaging by thermal radiation can reflect the difference in thermal information contained in different objects through grayscale intensities. Hence, infrared images can avoid the influences of illumination and occlusion, but the limited detail property of infrared images makes a big challenge to visual scene understanding. In contrast, visible images are captured by reflected lights, making them rich in textures while susceptible to external environments. The purpose of infrared and vision image fusion (IVIF) is to obtain an improved image that simultaneously contains both rich texture information and thermal radiation information of the target. From the perspective of vision-related applications, the promising fusion image can be used as a reliable upper source for subsequent visual tasks, which is crucial for good performance. In general, IVIF algorithms are mainly divided into two categories: traditional methods and deep learning-based methods.

Traditional approaches usually include multi-scale transform-based fusion algorithms, sparse representation-based fusion algorithms, subspace-based fusion algorithms, and hybrid fusion methods. A multi-scale transform model based on target enhancement was proposed in Ref.^2^. This algorithm uses the Laplace transform to decompose the aligned source image into high-frequency and low-frequency components, the fusion stage controls the infrared feature scaling by regularization parameters. Subsequently, the fusion image is reconstructed by an inverse Laplace transform. Briefly, these fusion algorithms decompose the source image into multidimensional features or transform the source image to other spectral spaces and then fuse the decomposed components using corresponding fusion strategies. The improvement in the fusion performance of these algorithms is mainly attributed to the precise decomposition manner and the efficient fusion strategy. To be specific, unsuitable decomposition will result in information loss on the source image. Besides, the complicated decomposition operators can lead to the degradation in fusion efficiency.

The deep learning-based fusion algorithms include convolutional neural network-based approaches and generative adversarial network-based methods. A neural network-based image fusion method is proposed in Ref.^3^, which builds a trained convolutional neural network to extract the features of the source image and then manually uses simple fusion rules to acquire the fusion image. The works^4–7^ exploit an encoder-decoder model as the backbone network, first exploiting the encoder for feature extraction, then performing the designed fusion rules, and finally reconstructing the fused image with a decoder. Moreover^8^ adopts a Res2Net-based encoder to extract the multi-scale features of the source images and employs spatial attention as the fusion rule, which can assign an effective weight map to fuse the meaningful features from source images. Meanwhile, generative adversarial network (GAN)^9^, with unique advantages in image generation, can approximate the distribution of real data arbitrarily in unsupervised means. Based on these characteristics, FusionGAN^10^ proposes a generative adversarial image fusion method. Using a single adversarial mechanism leads to unbalanced fusion effects and serious omissions of texture or edges in visible images. GANMcC^11^ introduces a generative adversarial mechanism to transfer image fusion into a multi-classification constrained problem, which can alleviate the imbalance trivially. However, the target edges in the fused images are blurred and the texture edge information cannot well be maintained. In view of the fusion strategies, most approaches use static fusion strategies designed by handcrafted means, which are not learnable and fail to perform fusion adaptively. As a result, this straightforward and fixed fusion strategy will further restrain the evolution of infrared and visible image fusion. To cope with this limitation, some adaptive fusion strategies have been proposed for optimal image fusion^12–14^. Concretely, RFN-Nest^12^ proposes a residual fusion network that performs multi-scale feature fusion, aimed at retaining more details in different modalities. An importance learning function is built for the adaptation strategy in multi-source domain adaptation^13^. Meanwhile, the learning-based feature fusion is developed for image super-resolution^14^.

**Figure 1:**
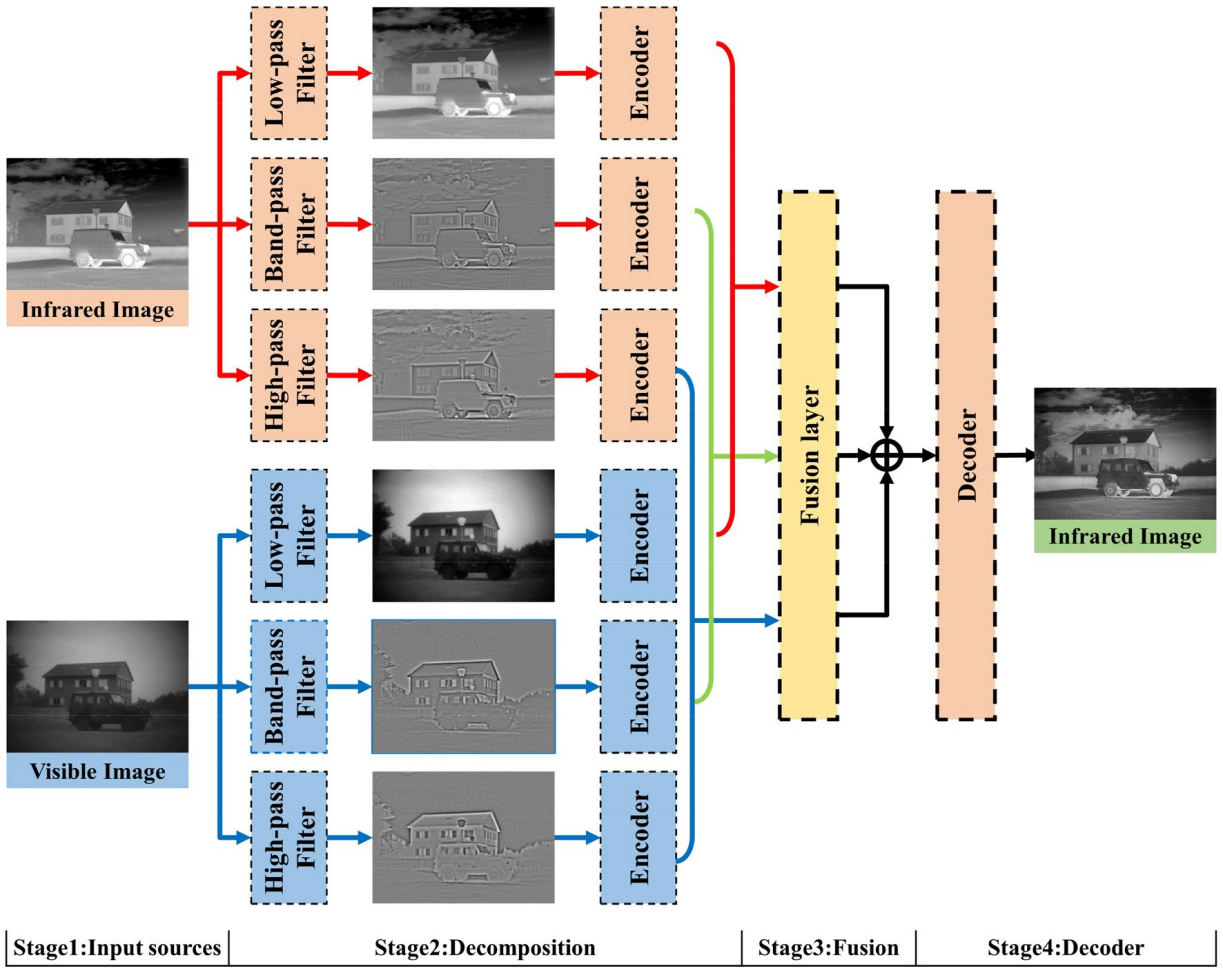
The framework of the proposed fusion network.

Recently, Zhao et al. proposed an efficient image fusion model (AUIF)^15^, which decomposes the source image into high-frequency and low-frequency bands through a traditional optimization model, and replaces the optimization iteration formula with a learnable neural network via algorithmic unrolling. Nevertheless, high-pass and low-pass filters, used in the AUIF network for two-scale decomposition of the source images, discard the medium-frequency information in images. Even if the cutoff frequency of the filter is well-designed, there is still some meaningful high-frequency and low-frequency information to be lost. In addition, the AUIF network utilizes the simple addition fusion strategy for high-frequency and low-frequency information, which also degrades the quality of the fused images. To alleviate these problems, we propose a network based on frequency-domain multiscale decomposition and interactive residual coordinated attention fusion. The framework of the proposed fusion network is shown in Fig. 1. Overall, the main contributions are summarized as follows:

1. A triple-branch feature extraction network in the frequency domain is developed to achieve the fine detail preservation from the perspective of multi-scale decomposition.
2. A dense network of residual gradients incorporating pyramidal segmentation attention (PSA) is designed to enhance the feature extraction from the medium-frequency information.
3. A learnable interactive residual coordinate attention fusion network (IRCAFN) is proposed to consider the feature correlation between infrared and visible images while building spatial feature attention across channels. The inability to adaptively fuse feature maps is a weakness of current methods^16–18^, which is overcome by IRCAFN, although many methods evade it by enhancing feature extraction capabilities.
4. A contrast loss function is leveraged to train the multiscale decomposition network for enhancing the complementarity of information at different scales. Moreover, a compensatory loss is introduced to train the efficient IRCAFN, aiming to balance the distinguishing information between infrared and visible images in the fused images.

Fusion results show that the fused images can retain rich detail information while focusing on the brightness of infrared targets. Our code for this model is available at https://github.com/shazong0526/ IRCAFusion.

## Related methodology

### Multi-scale decomposition

Multi-scale decomposition (MSD) is one of the classical operations for many IVIF algorithms. The main idea of IVIF based on MSD is to decompose the source image into a set of images according to specific rules, then fuse the decomposed images, and finally implement inverse MSD to reconstruct the fused images. Traditionally, the decomposition methods usually include pyramid transform^19^, discrete cosine transform^20^, nonsubsampled contourlet transform^21^ and bilateral filtering^22^, etc. In the meanwhile, the autoencoder (AE)-based approach is gradually gaining popularity and its structure consists of an encoder and a decoder. Data-driven AE-based networks offer great flexibility for image reconstruction. A representative fusion model is the dense block-based DenseFuse^17^. Such method relies on a large number of training samples from the MS-COCO dataset to train the autoencoder. The encoder corresponds to the MSD to extract effective features from the input image, while the decoder corresponds to the inverse MSD to reconstruct the image based on the encoded features^23,24^. Moreover, the loss of information in the multi-scale decomposition process is a key issue. Therefore, a low-loss multi-scale decomposition method is necessary to facilitate the effective information in the source image to be embodied in the fusion image. Based on this finding, we construct a low-loss AE network based on multi-scale decomposition in the frequency domain.

### Fusion rules

It is well known that the feature fusion strategy is a crucial influential factor in IVIF^25^. Most existing fusion strategies are simple traditional fusion rules, which cannot adaptively adjust the fusion weights according to specific image features. Briefly, little attention has been paid to the design of efficient fusion rules. Therefore, the choice of fusion strategies remains limited, including addition^18^, max^25^, average^26^, L1-normal^27^, etc. Recently, some deep learning-based fusion strategies have been proposed to solve the problem that the fusion effect is affected by the fusion rules. RFN-Nest develops learnable deep learning fusion networks that use residual fusion networks to fuse multi-scale features proposed by encoders^12^. Different information should be fused with corresponding fusion strategies to make full use of information from each scale. Besides, the information interaction between infrared and visible images should be emphasized when designing the fusion strategy, as infrared and visible images have some structural similarities.

To cope with these issues, the main motivation of this work is to explore an efficient learnable fusion rule for IVIF. Specifically, our fusion method introduces a new interactive residual coordinate attention fusion network (IRCAFN), which is employed in high-frequency feature fusion to retain more detailed information in fused images. Concretely, the coordinate attention weights of the two source images are weight interacted, and then the fusion of high-frequency features is accomplished by the framework using the residual connection structure. Experimentally, simple fusion conventional strategies are applied to fuse features at low and medium frequencies in our multi-scale decomposition network to keep a good tradeoff in the efficiency and computation complexity.

## Methods

In this section, we introduce a low-loss frequency domain decomposition image fusion network. The architecture of the proposed network is elaborated in Section "The framework of the proposed method". The two-stage training strategy is depicted in Section "The two-stage training strategy".

### The framework of the proposed method

**Figure 2:**
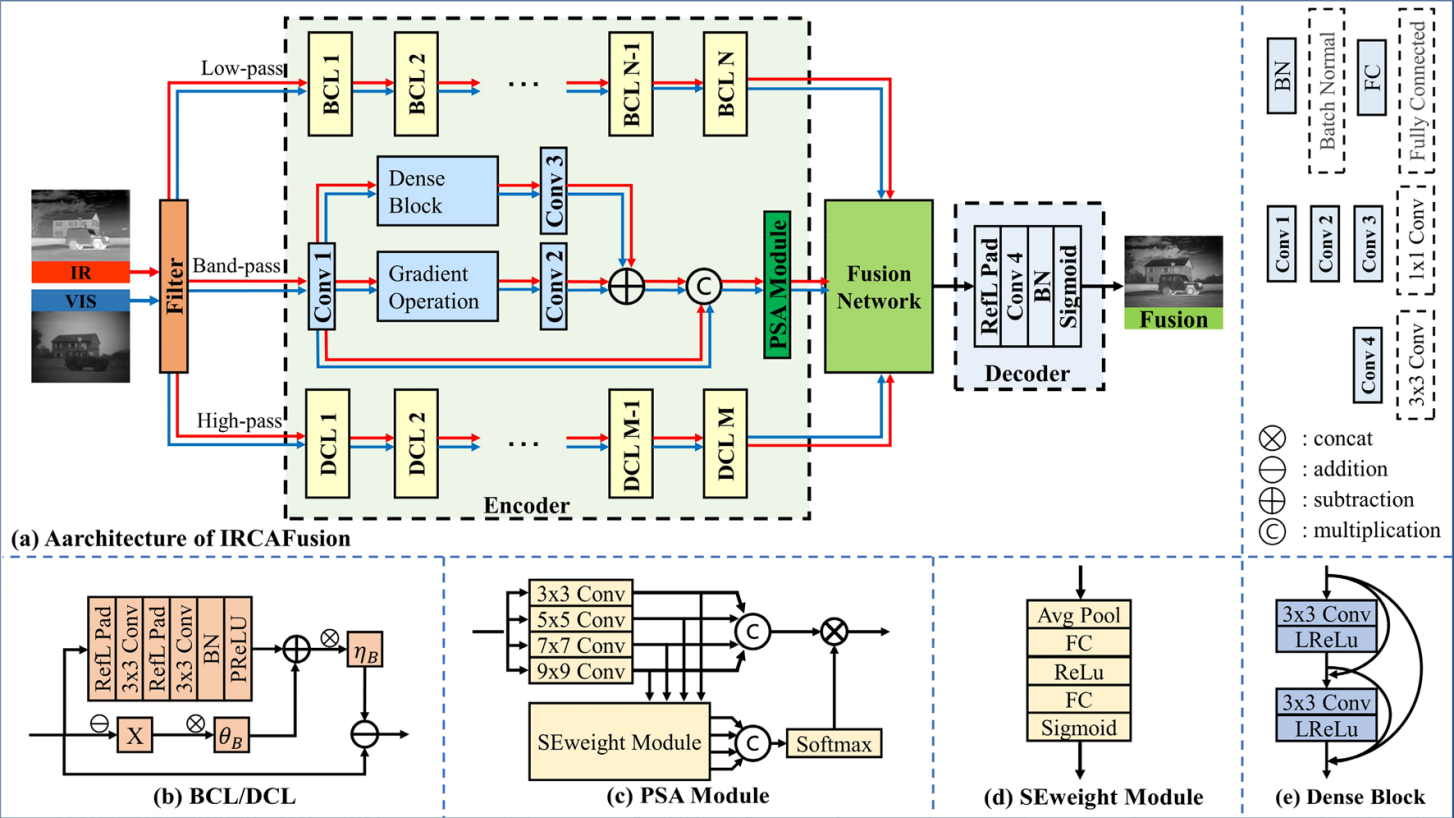
Illustration of the IRCAFN model. (**a**) The architecture of the IRCAFN. The Fusion network denotes the IRCAFN. (**b**) BCL and DCL with the same structure and different parameters. (**c**) The architecture of the PSA module. (**d**) The architecture of the SE weight module. (**e**) The architecture of the Dense Block.

Our network consists of three parts: the encoder, the interactive residual coordinate attention fusion network (IRCAFN), and the decoder. The detailed framework is shown in Fig. 2.

Our network begins with a multiscale decomposition of the image, where the source image is decomposed into the frequency domain using different filters for low-loss multiscale representation. The high-frequency detail information and low-frequency base information are extracted using the high-pass and low-pass filters, respectively. In particular, we set the cut-off frequency of the band-pass filter according to the settings of the high-pass and low-pass filters. The specific bandpass filters are introduced for both high and low-frequency information, to extract useful information that has been disregarded.

Then, the AUIF network is embedded to extract informative features. The feature extraction component of the AUIF network consists of N convolutional layers, called base convolutional layer (BCL) or detail convolutional layer (DCL), and each convolutional layer is converted from the traditional optimization algorithms by algorithmic unrolling. The basic structure of AUIF is shown in Fig. 2. The reflection-padded structure is to prevent artifacts at the image edges. The batch normalization layer and parametric rectified linear unit (P-ReLU) can enhance the feature extraction capability^15^.

Especially, for medium-frequency information, we employ a dense residual gradient network (DRGN) to extract features. Additionally, the additional PSA attention module is integrated to suppress redundant information.

Finally, this work builds an IRCAFN network to fuse the high-frequency features of infrared and visible images and designs a conventional module to fuse the low-frequency and medium-frequency features of images. As for the decoder, its function is to reconstruct the fusion image. The decoder consists of a 3 × 3 convolution unit, batch normalization layer, and the sigmoid function. In other words, the input of the encoder is the fused features and its output is the reconstructed image. The following parts will introduce the medium-frequency feature representation and the interactive residual coordinate attention fusion network.

#### The dense residual gradient network (DRGN)

**Figure 3:**
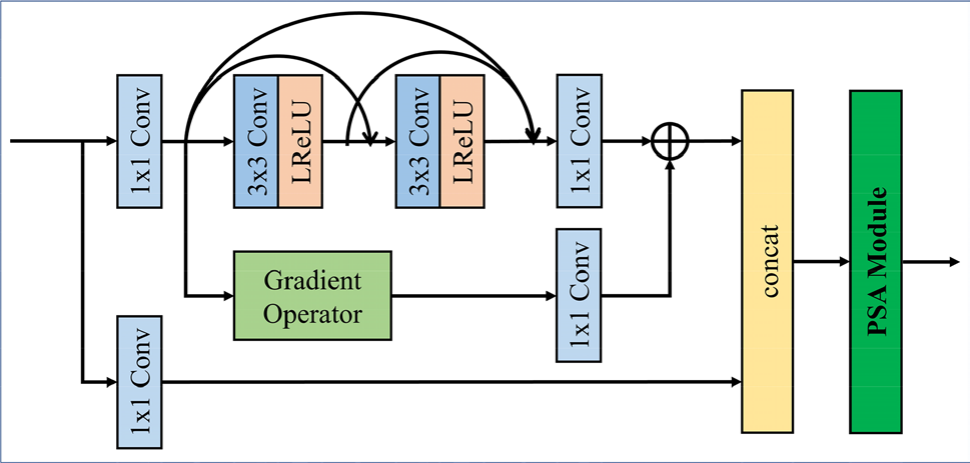
The structure of dense residual gradient network (DRGN).

The motivation of this work is to build more efficient and effective feature networks. Therefore, A new DRGN incorporating a pyramid squeeze attention (PSA) module is proposed. There are no ideal high-pass and low-pass filters, which makes it difficult for the realization of the medium-frequency components. The specific design of DRGN is illustrated in Fig. 3. DRGN is a targeted variant of the gradient residual dense block (GRDB)^28^, which consists of three feature extraction branches. To extract useful information in the medium-frequency band, this component exploits dense connections in the innermost layer. The residual branch with an integrated gradient operator helps to extract the high-frequency detail information from the medium-frequency content. In particularly, considering the small amount of useful information in the medium-frequency, some informative features may be omitted in multiple convolution operations. Consequently, we introduce an additional branch of raw information in the outermost layer, which constitutes the external dense connection structure. The structure of the PSA module is shown in Fig. 2c. The PSA module can learn diverse multi-scale feature representations and adaptively re-compare cross-dimensional channel attention weights^29^. Briefly, the DRGN with PSA can capture more informative detail in the medium-frequency for subsequent fusion, which contributes to less information loss.

#### Interactive residual coordinate attention fusion network (IRCAFN)

**Figure 4:**
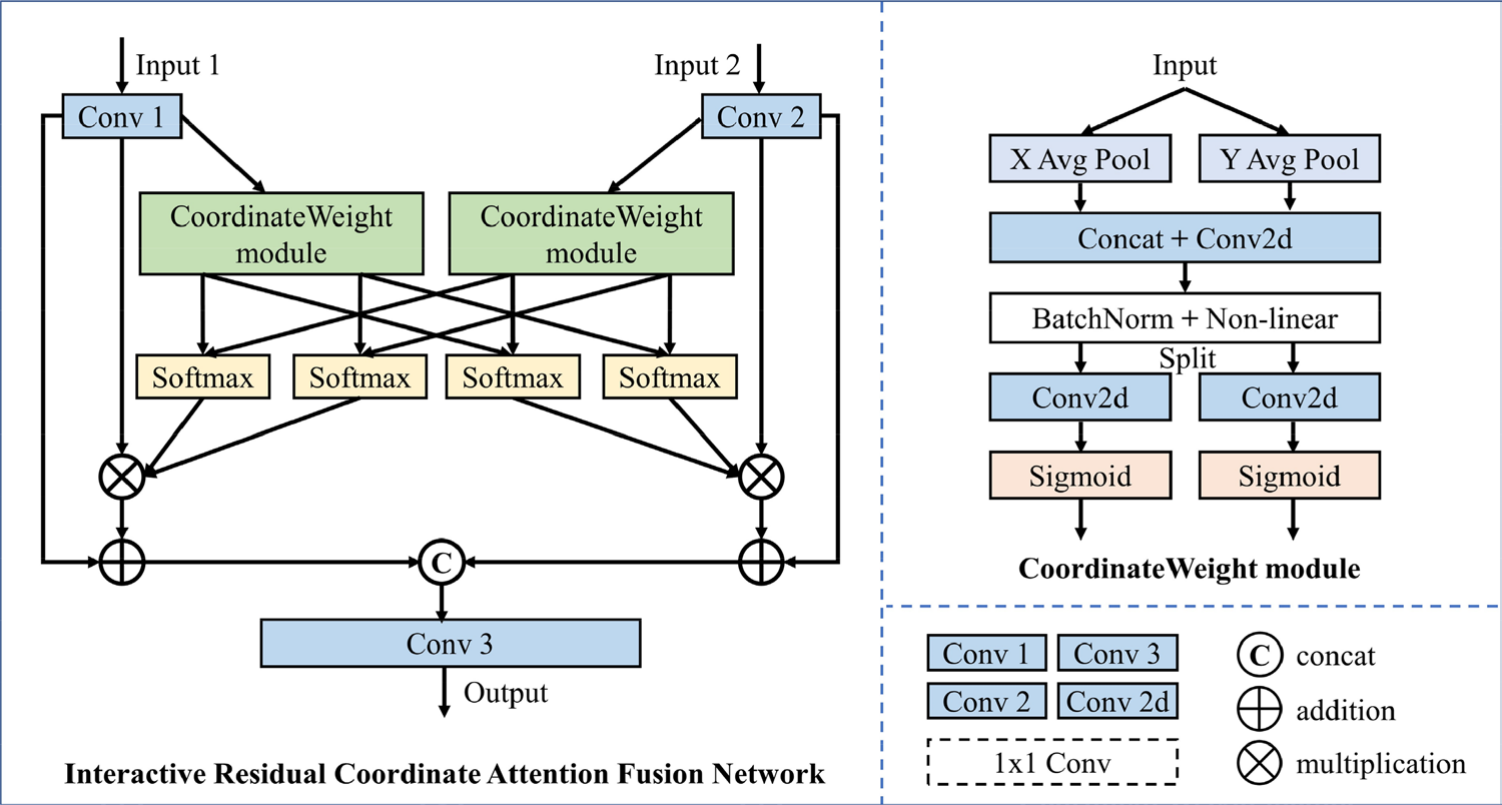
The structure of the Interactive Residual Coordinate Attention Fusion Network (IRCAFN).

The IRCAFN is based on the prevalent residual structure. To be specific, the coordinate attention module is applied in this residual structure, instead of traditional convolution operation. The detailed structure of IRCAFN is shown in Fig. 4.

Motivated by the mechanism in^30^, we obtain the attention weights for the horizontal and vertical directions separately for the high-frequency information feature maps of the source images. This method achieves cross-channel information interaction of intra-image features and establishes a long-distance correlation of intra-image features. Then, the attention weights of horizontal and vertical directions of different source images are adjusted correspondingly. Therefore, it can acquire learnable weight interaction between features and structures across different source images. Finally, the high-frequency information of the source image is multiplied with the corresponding weights and then summed with the source image to obtain the fused high-frequency features.

### The two-stage training strategy

The feature extraction and reconstruction of the codec network play vital roles in the image fusion. Consequently, we applied a two-stage training strategy to improve the fusion efficacy.

#### Training of the auto-encoder network

**Figure 5:**
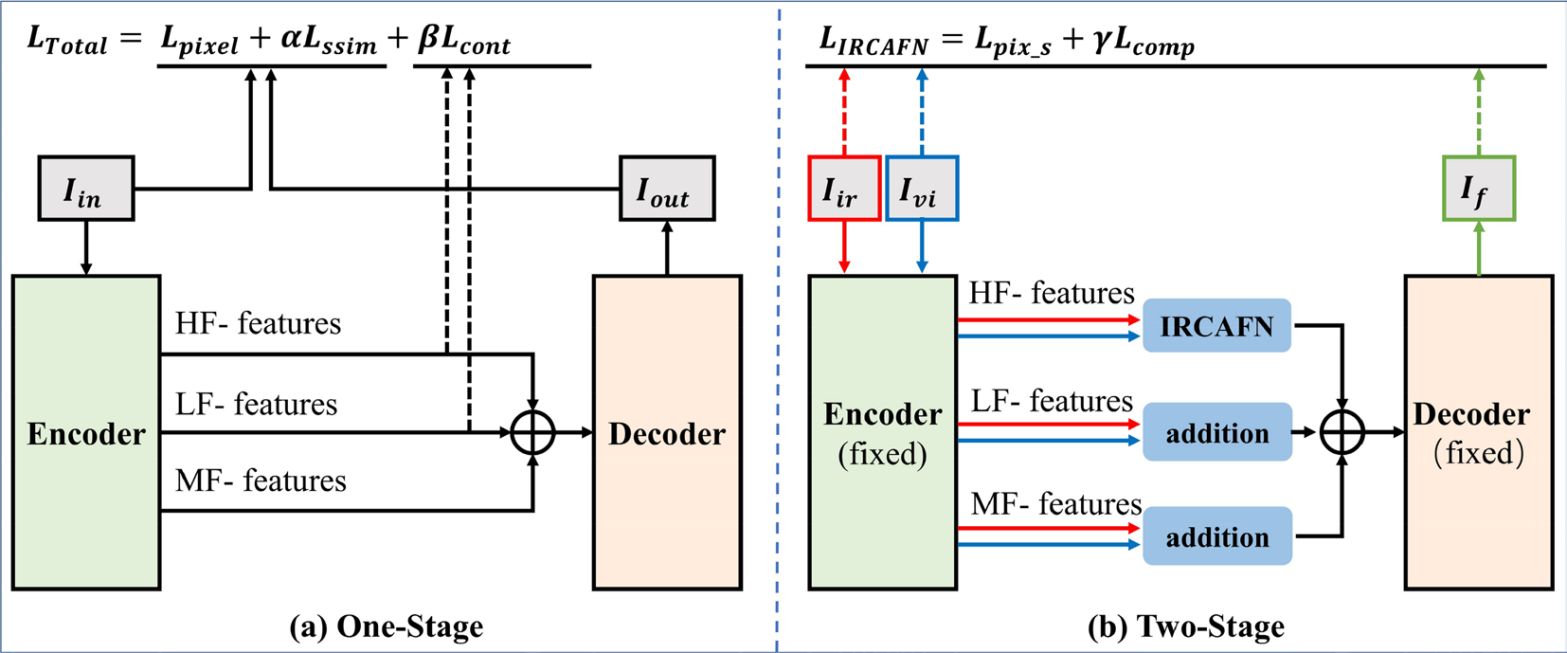
The structure of the two-stage training.

The encoder-decoder network is shown in Fig. 5a. The input image is decomposed to obtain high-frequency detail features, medium-frequency features, and low-frequency base features. All features are adaptively summed up and sent to the decoder for the final reconstruction.

The encoder network is trained using the loss function ${L}_{Total}$ defined as follows,

1 $$ L_{Total} = I_{pixel} + \alpha I_{ssim} + \beta I_{cont} $$

2 $$ L_{Total} = \left\| {I_{out} - I_{in} } \right\|_{F}^{2} + \alpha \left[ {1 - SSIM\left( {I_{out} ,I_{in} } \right)} \right] + \beta COS\_SIM\left( {I_{HF} ,I_{LF} } \right) $$

where $I_{pixel}$,$I_{ssim}$,$I_{cont}$ denote the pixel loss, the structure similarity loss, and feature contrast loss.$I_{out}$,$I_{in}$ denote the input images and reconstructed images.$I_{HF}$,$I_{LF}$ represents the encoded high-frequency feature and low-frequency feature. $\left\| \cdot \right\|_{F}^{2}$ is the Frobenius norm, $SSIM\left( , \right)$ is the structural similarity index which quantifies the structural similarity between the output image and input image^31^. $COS\_SIM\left( , \right)$ is the cosine similarity used to enhance the complementarity of the encoded high-frequency and low-frequency information.$\alpha$, $\beta$ are the hyperparameters for balancing different items’ weights.

#### Training of the IRCAFN

In the second stage, we employ a learnable IRCAFN combined with a traditional summation rule as the final fusion strategy. The architecture of the training fusion network is shown in Fig. 5b. In the medium-frequency and low-frequency information of the image, we use the traditional fusion rule of addition. For the crucial fusion strategy of high-frequency details, a learnable IRCAFN is developed to accomplish fusion.

In the training process, we adopt a mutually compensating loss function that uses the Frobenius norm to bound the luminance loss of the fused image to the visible and infrared images. However, the Frobenius norm only provides a coarse-grained distribution constraint for model learning, which will enlarge the intensity difference. As the Sobel gradient operator can measure the fine-grained texture information of an image and the L1-norm has the good sparsity for detail preservation^28^, additional L1-norm loss on Sobel gradient operator is compensated for the loss of visible texture. In short, considering the compensation of different constraints, the joint loss $L_{IRCAFN}$ can simultaneously gain the good intensity distribution and detail information with the assistance of the Sobel gradient operator. $L_{IRCAFN}$ defined as follows,

3 $$ L_{IRCAFN} = I_{pix\_s} + \lambda I_{comp} $$

4 $$ L_{IRCAFN} = \left\| {I_{f} - I_{vi} } \right\|_{F}^{2} + \delta \left\| {I_{f} - I_{ir} } \right\|_{F}^{2} + \lambda \left\| {\nabla I_{f} - \nabla I_{vi} } \right\|_{1} $$

where $I_{pix\_s}$ denote the total pixel loss from infrared and visible images, and $I_{comp}$ denote the compensation loss between the fused image and the visible images. $I_{f}$,$I_{ir}$,$I_{vi}$ denote the fusion image, infrared image, and visible image, $\left\| \cdot \right\|_{F}^{2}$ is the Frobenius norm. $\nabla$ denotes the Sobel gradient operator^28^, which measures the gradient difference between the fused image and the visible images. $\left\| \cdot \right\|_{1}$ is the L1-norm. $\delta$ and $\lambda$ are balance hyperparameters.

## Experiments

In this section, comprehensive experiments are conducted on prominent databases to verify the effectiveness and superiority of the IRCAFN. All experiments are conducted with Pytorch (version 1.7.1) on a computer with Windows 10 Operating System, Intel Core i5-10400F Processor, 16 GB Memory and GeForce RTX3070 GPU.

### Datasets and metrics

The FLIR dataset is chosen as the training set for both stages. In the test phase, we used TNO^32^, FLIR^33^, and NIR^34^. The details are shown in Table 1.

**Table 1:** The details of experimental datasets.

Dataset	Train			Test
	FLIR-Train	TNO	FLIR-Test	Country-NIR
Illumination	Day&Night	Night	Day&Night	Day
# Image pairs	180	40	40	52

Six quality assessments are chosen for performance evaluation: entropy (EN), standard deviation (SD), spatial frequency (SF), visual information fidelity (VIF), average gradient (AG), and the sum of the correlations of differences (SCD). The goal of image fusion is to combine images from several scenes to create a single, more informational, and expressive image. The fused image's pixel grayscale difference and gradient distribution are measured using SD and SF, which are directly related to the image's contrast and sharpness. EN and AG are used to define the fused image's amount of information and detail information. The complementary information gathered from the various source images is essential to the success of image fusion. Quantifying the amount of information transferred from each source image to the fused image, SCD is the sum of the source image and fused image difference image to source image correlation. VIF is used to quantify the information fidelity between the fused image and the source image. A higher value of VIF indicates that the fused image is more in line with the human eye's visual perception system. Overall, the increase in the value of the above metrics can represent the improvement in image fusion effects. More details of the definition of those metrics can be found in the reference^1^.

### Implementation details and network configuration

In the first training stage, the batch size and epoch are set to 30 and 120, respectively. The learning rate is 1e−2 for the first 96 epochs and it is decreased to 1e−3 for the rest epochs. α and β in Eq. (1) are set to 4.8 and 0.09. For the learnable parameters $\eta$ and $\theta$ in high-frequency and low-frequency feature extraction branches, we set them to 0.1 and 0.03, referring to the AUIF network^15^. As for the number of repeated BCL/DCL units in the high-frequency and low-frequency feature extraction branches, we experimentally verified that $H_{n}$ and $L_{n}$ are 7 and 12. After the training is completed, we use the simple addition fusion rule for fusion to verify the fusion effect.

In the second training stage, the batch size and epoch are set to 18 and 150. The learning rate is 1e−2. The hyperparameters $\delta$ and $\lambda$ of Eq. (4) are set to 3.4 and 0.5. We employ the pretrained encoder-decoder model generated in the first training phase, using IRCAFN as the fusion strategy for the high-frequency detailed features. As for the low-frequency and medium-frequency feature fusion strategies, we choose a simple addition fusion strategy.

### Experimental results

#### Effectiveness of the scale decomposition

##### Qualitative analysis

**Figure 6:**
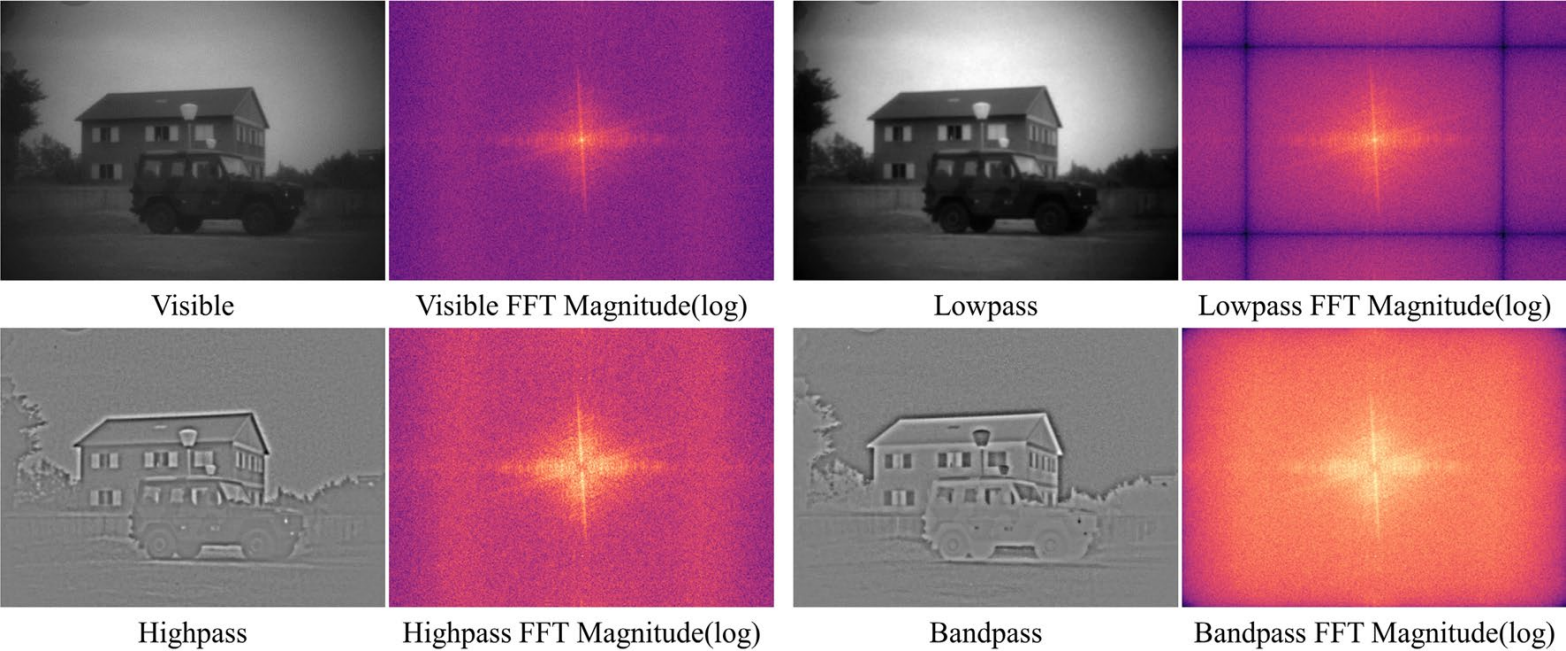
Frequency domain multi-scale decomposition images and corresponding spectrograms.

The image in the scale decomposition stage of the frequency domain and the corresponding spectrogram are shown in Fig. 6. Figure 6 directly reflects the result of feature decomposition, which decomposes the source image into high-frequency, medium-frequency, and low-frequency bands. There is some valuable information in the medium-frequency information, which is easily seen in the resultant images and spectrum plots of the bandpass filter.

##### Quantitative analysis

To objectively evaluate the effectiveness of the scale decomposition, the results of the AUIF method were used as the experimental baseline. Therefore, an addition fusion strategy is employed on the same TNO dataset, the results are listed in Table 2. $L_{n}$,$M_{n}$,$H_{n}$ represent the number of convolution units of high-frequency, medium-frequency, and low-frequency feature decomposition networks, respectively. DRGN + PSA denotes that the network structure consisting of DRGN and PSA is used for the medium-frequency feature extraction branch. The first row in Table 2 presents the replication results of AUIF. From the first and second rows in Table 2, almost all evaluation metrics were improved to varying degrees after supplementing the information on medium-frequency characteristics. Especially, the common improvement of EN, SD, and SF evaluation indexes indicates better definition and richer information in fusion images. This finding verified the practicability of our proposed decomposition network with three scales in the frequency domain.

**Table 2:** Results of frequency domain multi-scale decomposition network experiment.

$L_{n} - M_{n} - H_{n}$	EN	SD	SF	VIF	AG	SCD
10-00-10 (AUIF)	6.9438	43.4466	11.0526	0.8113	4.1458	1.8602
10-10-10	6.9542	43.1086	11.6106	0.8144	4.3356	**1.8664**
08-10-10	6.9278	44.6968	11.6489	0.8082	4.2762	1.8380
10-08-10	6.9300	44.9980	**11.6939**	0.8093	**4.3420**	1.8390
10-10-08	6.9171	42.1906	11.1482	0.8022	4.1633	1.8536
10-10-12	6.9527	41.7335	11.0188	0.8143	4.1898	1.8655
07-10-12	**6.9889**	**45.4409**	11.3499	**0.8231**	4.2786	1.8622
07-08-12	6.9658	44.9941	11.3766	0.8153	4.2966	1.8580
07-(DRGN + PSA)-12	*7.0833*	*45.5016*	*12.7390*	*0.8422*	*4.8324*	*1.8839*

The optimal and second values are emphasized in italics and bold respectively.

Different scales of information should alter to the corresponding feature extraction network. Accordingly, comparison experiments on feature extraction networks are conducted in the framework of setting the frequency domain scale decomposition.

First, the number of convolutional units in each feature decomposition network make variable impacts on the fusion results. The results show that the best fusion effect is achieved when the number of convolutional units in the high-frequency, low-frequency, and medium-frequency feature extraction branches satisfies $L_{n} < M_{n} < H_{n}$. $L_{n}$,$M_{n}$,$H_{n}$ represent the number of convolution units of high-frequency, medium-frequency and low-frequency feature decomposition networks, respectively.

Furthermore, a feature extraction network combining PSA and DRGN is utilized as the medium-frequency feature extraction branch. Based on the experimental results in Table 2, the numbers of convolutional units of the high-frequency and low-frequency information extraction networks are set to 7 and 12. The proposed network obtains the maximum boost in all metrics. The proposed network obtains the biggest improvement in all metrics, further confirming the validity of the three-scale decomposition for image fusion.

Subsequently, relevant ablation experiments are conducted to confirm the superiority of DRGN and PSA. The results of the ablation study are given in Table 3. When DRGN is used only in the medium-frequency feature extraction branch, the evaluation indexes of SF, AG, and SCD are improved, which indicates a higher definition of the image. With the addition of the PSA module, nearly all metrics are enhanced, reflecting the PSA module's ability to suppress interfering features in the medium-frequency domain.

**Table 3:** Ablation experiments of DRGN and w/o denote WITHOUT.

$L_{n} - M_{n} - H_{n}$	DRGN (w/o PSA)	PSA	EN	SD	SF	VIF	AG	SCD
7–10-12			6.9610	*45.5780*	11.4180	0.8150	4.270	1.851
7–10-12		√	**7.013**	45.462	11.528	**0.832**	4.336	**1.870**
7-DRGN-12	√		6.923	44.202	**11.840**	0.804	**4.377**	1.852
7-DRGN-12	√	√	*7.083*	**45.502**	*12.739*	*0.842*	*4.832*	*1.884*

The best and second values are marked in italics and bold respectively.

**Figure 7:**
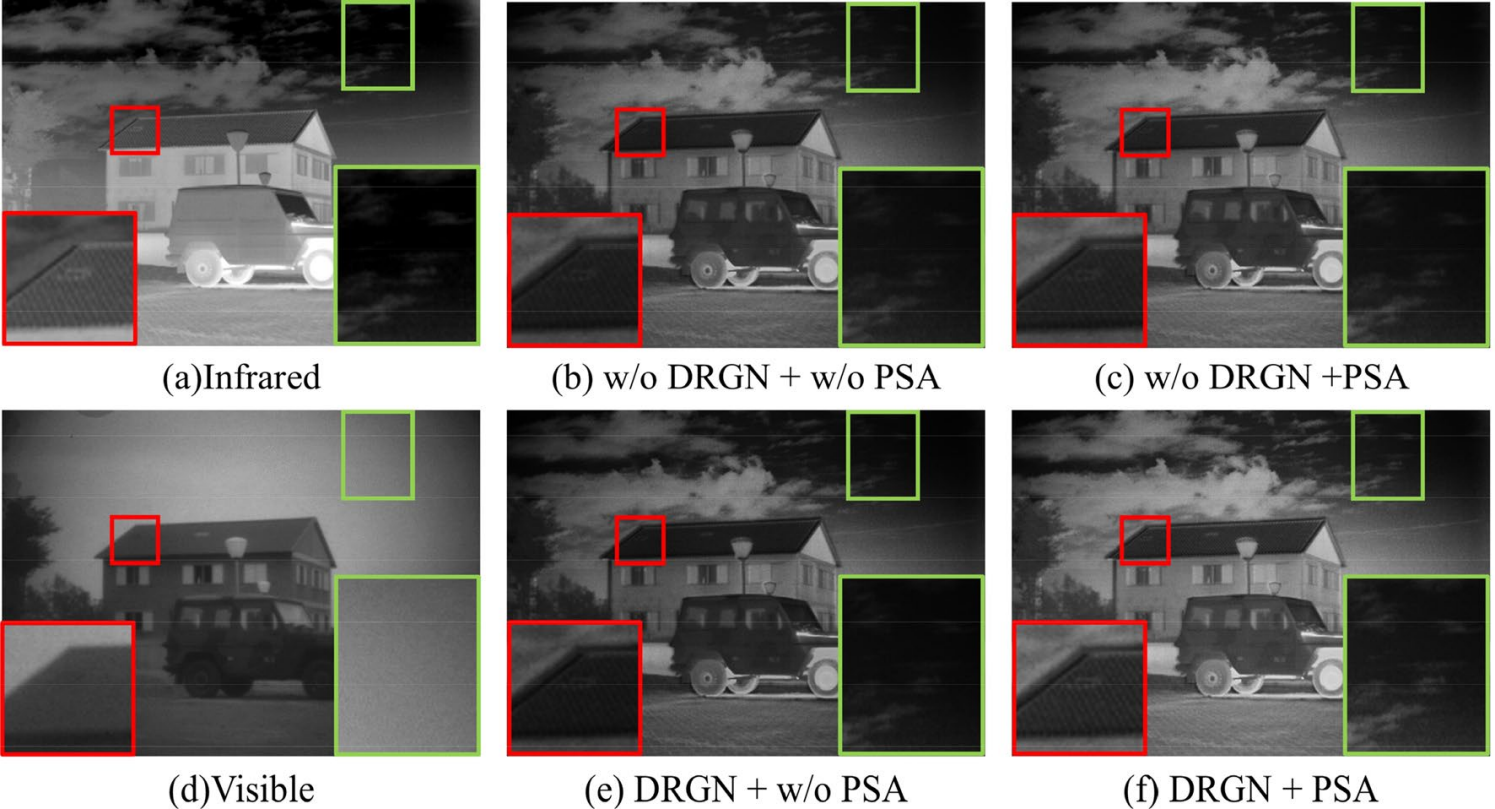
Test results of the ablation experiment for DRGN. The red and green boxes mark and magnify the selected area.

The similar conclusion can be drawn from the comparison of qualitative results in ablation experiments. The results are shown in Fig. 7, where Fig. 7a is the infrared image, Fig. 7b is the visible image, Fig. 7c is the fusion image without DRGN module, Fig. 7d is the fusion image without DRGN and PSA modules, Fig. 7e is the fusion image without the PSA module, Fig. 7f is the fusion image with DRGN and PSA modules. Compared with other fusion results, the relative luminance intensity in Fig. 7c,f is enhanced, which enables tiny details of low illumination to be preserved. Therefore, it illustrates the excellent effectiveness of the DRGN network for extracting mid-frequency features. In addition, Fig. 7f has the clearest detail preservation, such as the chimney on the house and the tiny clouds, which shows the PSA module suppresses the redundant information in the middle-frequency feature to strengthen the quality of the fused images. Overall, it can be inferred that the optimal fusion can be obtained by combining DRGN and PSA.

#### Effectiveness of two-stage training strategy

In the first stage of training, we use traditional $I_{pixel}$ and $I_{ssim}$ loss functions that improve the feature extraction and image reconstruction capabilities of the proposed model. Since filtering operations cannot be trained to improve feature decomposition in the multi-scale decomposition stage. To fully describe the complementarity of high-frequency and low-frequency features, the proposed model is notably supplemented with a cosine similarity constraint as $I_{cont}$. In the second training stage, $I_{pix\_s}$ constrains the pixel loss of the fused image. Furthermore, a compensation loss $I_{comp}$ based on the Sobel gradient operator is integrated to reduce the loss of detail texture in the fused image and achieve a balance between infrared content and visible information.

**Figure 8:**
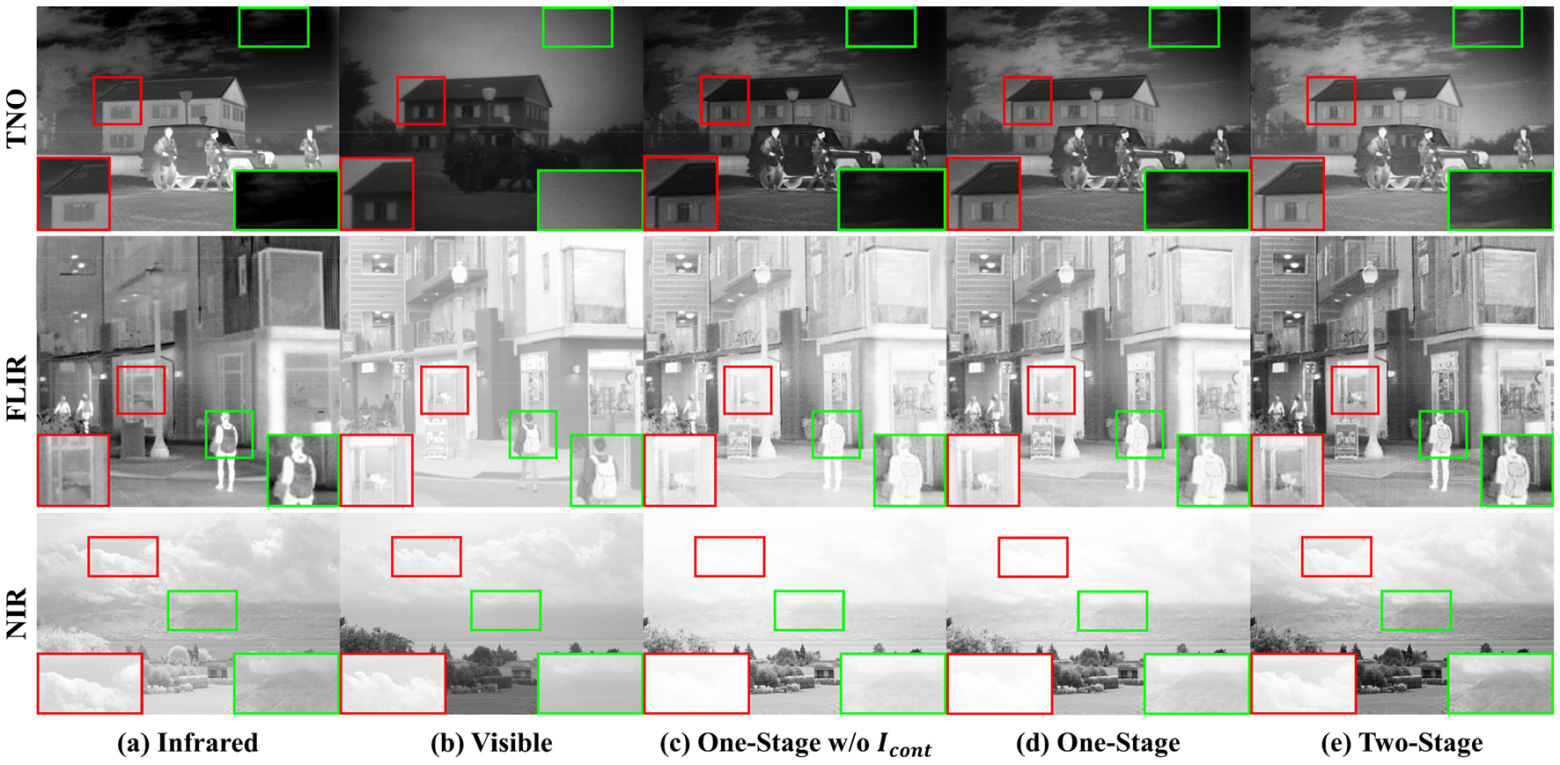
Test results of two-stage training. Red and green boxes mark and enlarge the selected area.

To present the feasibility of the two-stage of training strategies, the quantitative experimental results are given in Table 4, and the qualitative experimental comparison results are shown in Fig. 8.

**Table 4:** Ablation experiment of two-stage training strategy. Optimal values are marked in bold.

One-Stage		Two-Stage		Dataset: TNO					
$I_{pixel} + I_{ssim}$	$I_{cont}$	$I_{pix\_s} + I_{comp}$		EN	SD	SF	VIF	AG	SCD
√				7.083	45.502	12.739	0.842	4.832	1.884
√	√			7.099	**48.508**	12.384	**0.856**	4.718	1.874
√	√	√		**7.126**	45.610	**13.083**	0.836	**5.020**	**1.886**
				Dataset: FLIR					
√				7.320	49.402	14.963	0.723	5.678	1.799
√	√			7.504	54.553	15.876	**0.749**	6.150	1.886
√	√	√		**7.546**	**54.999**	**17.712**	0.747	**6.794**	**1.895**
				Dataset: NIR					
√				7.335	62.032	27.686	1.033	9.606	1.592
√	√			7.457	64.386	26.633	**1.074**	9.294	1.730
√	√	√		**7.492**	**66.609**	**28.711**	1.071	**9.908**	**1.755**

##### Qualitative analysis

For the test results of the TNO dataset in Table 4, a comparison between the first and second rows illustrates the improvement in meaning information and fidelity of the fused images with the addition of contrast loss. More importantly, fused results for the FLIR and NIR datasets show a considerable improvement in almost all evaluation metrics. The second stage test comparison results are shown in rows 2 and 3 of the data section for each dataset in Table 4. We can see that almost all evaluation metrics were enhanced for all three datasets, which indicates that the fused images provided richer information and a clearer visual effect. In summary, the results of the ablation experiments on loss functions demonstrate the effectiveness of the integration of contrast loss, which promotes the feature disentanglement of high-frequency and low-frequency information. Overall, the quantitative experimental results demonstrate the reasonability of the two-stage training strategies.

##### Quantitative analysis

The test results in Fig. 8c,d exhibit missing cloud shapes and missing backpack detail textures. The results illustrate that both visible and infrared images appear to have different degrees of information loss after the one-stage training. In contrast, the results of two-stage training in Fig. 8e can retain more detailed information from both visible and infrared images. On the other hand, the test results of the NIR dataset in Fig. 8e illustrate that the two-stage training results can enhance the contrast of the fused images, which makes the textures of the distant mountains clearer. The main reason is that in the multiscale decomposition stage, the introduction of the medium-frequency information brings a certain amount of redundant information while increasing the expected useful information. Notably, some important high-frequency detail features may be overwhelmed by redundant information. The comparison results explain the trained IRCAFN can enhance detailed information while suppressing redundant information. Hence, this work develops a two-stage training strategy. In the first stage, the multi-scale decomposition, feature extraction, and reconstruction capabilities of the AE network are trained. In the second stage, the IRCAFN network is trained as a fusion network for high-frequency detail information. The effectiveness of the two-stage training strategy is validated by the visualized results in Fig. 8.

#### Experiments on the fusion strategy

As the same amount of redundant information is introduced when adding medium-frequency branches, insufficient enhancement for the NIR/FLIR dataset can be gained when using DRGN and PSA medium-frequency extraction networks. Moreover, the overall image brightness of the NIR and FLIR dataset is higher than that of the TNO dataset, and a direct summation fusion strategy will cause the image brightness to be too large, which affects the fusion effect. This phenomenon illustrates that a suitable fusion strategy plays an essential role in image fusion. The traditional fusion algorithms, which are commonly used, as shown in Table 5, are applied to find the optimal fusion strategy for different information extraction branches.

**Table 5:** Typical fusion algorithms. $I_{f}$, $I_{ir}$,$I_{vi}$ are the feature maps of infrared image, visible image, and fusion image, respectively.

Fusion Strategy	Definition
addition	$I_{f} = I_{ir} + I_{vi}$
max	$I_{f} = max(I_{ir} ,I_{vi} )$
average	$I_{f} = avg(I_{ir} ,I_{vi} )$
L1-norm	$I_{f} = L1(I_{ir} ,I_{vi} )$

##### Qualitative analysis

**Figure 9:**
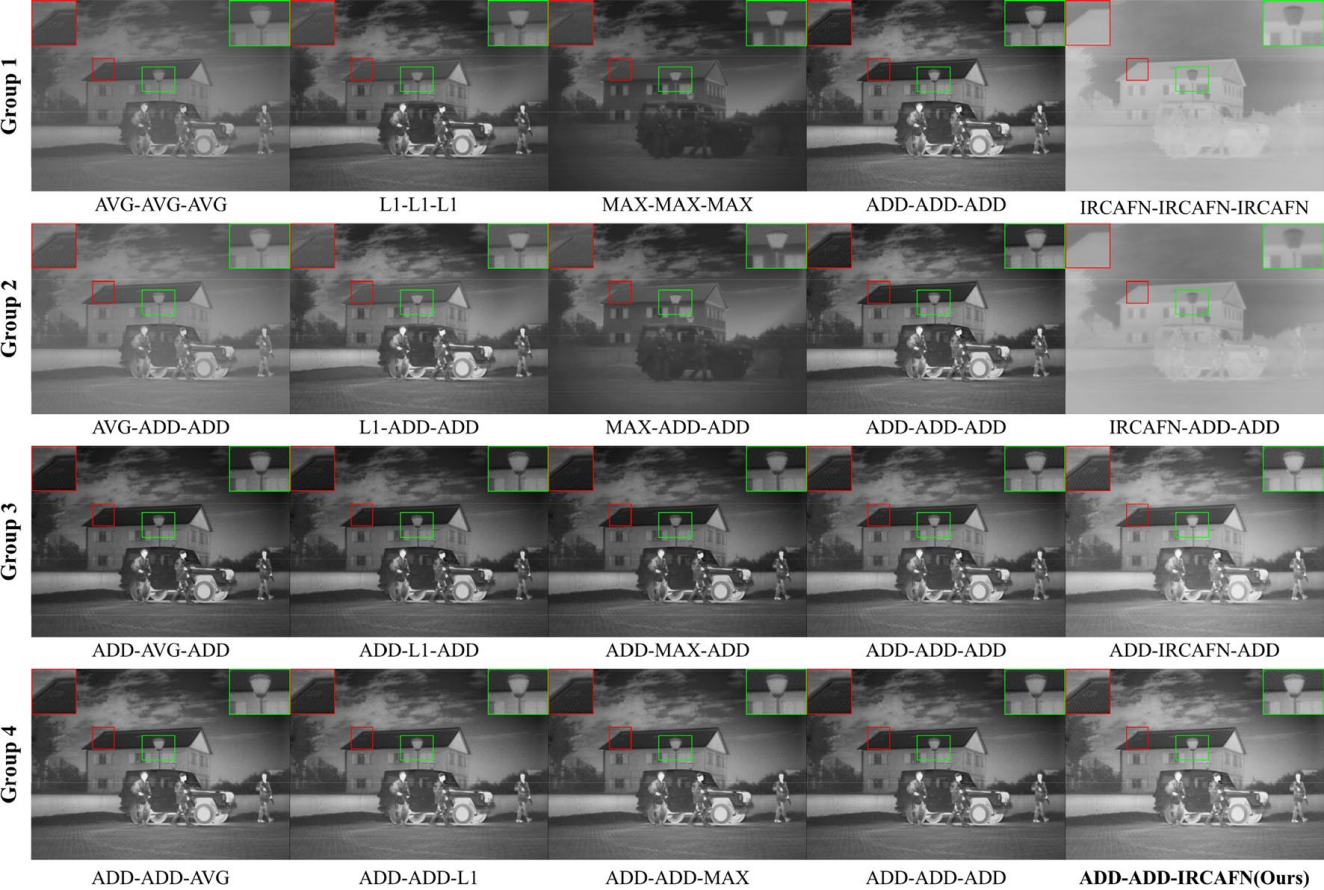
Comparison of the results of fusion strategies. Red and green boxes mark and enlarge the selected area.

The essence of the fusion strategy is using specific weighting elements to fuse the information from different source images. Clearly, the results of group 1 in Fig. 9 show that only the L1-normal and addition fusion strategies obtain sufficient information from the infrared and visible images. Unfortunately, the L1-normal method results in losing the detail content of the roof when the same fusion strategy is applied to features of different frequencies. Therefore, further experiments are based on the addition fusion strategy. Group 2 shows that, with low-frequency features, only the addition fusion strategy can balance the fused image with the appropriate brightness while retaining more detailed information. The enlarged detail image in Group 3 confirms the importance of the addition fusion strategy for the mid-frequency feature. The results exhibit optimal image contrast and detail retention features when using the proposed IRCAFN as a high-frequency detail feature fusion strategy. Therefore, the experimental results achieved the best visual results when the fusion strategies of IRCAFN, addition, and addition were applied to the high-frequency, medium-frequency, and low-frequency features, respectively.

##### Quantitative analysis

Our experiments are based on the additive strategy, which has been proven to be a suitable fusion strategy for such feature decomposition by the AUIF network. From the first set of results for the three datasets in Table 6, the addition fusion strategy performs best in the three datasets when the same fusion strategy is implemented for different information. It is reasonable that different fusion strategies should be adopted for different scales. A series of experiments are presented and the results are shown in Table 6. As shown in Table 6, the test results in three datasets were evaluated comprehensively, and the FLIR dataset and the NIR dataset achieved an overwhelming lead when the addition fusion strategy is applied to the low and medium frequency information, while the TNO data achieved an average index.

**Table 6:** Comparison of experiments of fusion methods. The best and second values are marked in italics and bold respectively.

	Fusion strategy	EN	SD	SF	VIF	AG	SCD
Dataset: TNO							
Group-1	avg-avg-avg	6.4446	27.1876	7.6863	0.6912	2.9569	1.6293
	L1-L1-L1	6.8766	40.3483	9.6348	0.8193	3.7040	1.7353
	max-max-max	6.1390	23.6213	7.2180	0.7782	2.6898	1.1783
	add-add-add	7.0833	*45.5016*	**12.7390**	0.8422	**4.8324**	**1.8839**
	IRC-IRC-IRC	6.5927	31.3066	8.4547	0.1120	3.2880	0.0216
Group-2	avg-add-add	6.5118	27.9558	8.1110	0.7097	3.1690	1.6563
	L1-add-add	6.9354	40.7883	9.9588	0.8364	3.8826	1.7516
	max-add-add	6.3127	26.1534	8.0958	0.8393	3.0422	1.2103
	IRC-add-add	6.5308	29.6045	7.1804	0.1415	2.6923	0.0567
Group-3	add-avg-add	**7.0858**	45.3538	12.5440	**0.8423**	4.7475	1.8823
	add-L1-add	7.0853	45.3420	12.5364	0.8421	4.7441	1.8823
	add-max-add	7.0841	45.3150	12.5147	0.8419	4.7371	1.8820
	add-IRC-add	6.9947	42.3011	11.4793	0.8006	4.2380	1.8214
Group-4	add-add-avg	7.0150	44.8489	12.5221	0.8270	4.6954	1.8734
	add-add-L1	7.0150	44.8492	12.5222	0.8270	4.6954	1.8734
	add-add-max	7.0144	44.8260	12.5181	0.8268	4.6935	1.8732
Ours	add-add-IRC	*7.1047*	**45.4364**	*12.8173*	*0.8455*	*4.8847*	*1.8879*
Dataset: FLIR							
Group-1	avg-avg-avg	6.9067	33.3131	10.4493	0.6676	4.0699	1.4569
	L1-L1-L1	7.2013	42.4035	12.8600	0.7558	4.9802	1.5548
	max-max-max	7.0175	38.3737	11.4534	**0.7931**	4.3142	0.9967
	add-add-add	7.3197	49.4016	14.9627	0.7233	5.6780	1.7989
	IRC-IRC-IRC	6.4366	29.3023	9.4984	0.0884	3.1873	0.2702
Group-2	avg-add-add	6.8795	32.5316	10.3117	0.6666	4.0630	1.4267
	L1-add-add	7.1361	40.8838	12.3817	0.7458	4.8295	1.5296
	max-add-add	7.0887	40.0901	12.0683	*0.8120*	4.5792	1.0103
	IRC-add-add	6.3304	26.7248	7.8696	0.1185	2.6432	0.2153
Group-3	add-avg-add	7.2733	48.3193	14.3863	0.7166	5.4296	1.7756
	add-L1-add	7.2737	48.3181	14.3860	0.7163	5.4280	1.7757
	add-max-add	7.2767	48.3721	14.4247	0.7162	5.4405	1.7770
	add-IRC-add	6.8055	38.8326	11.0031	0.6374	3.9525	1.4964
Group-4	add-add-avg	7.4560	52.6341	16.1879	0.7412	6.2015	1.8668
	add-add-L1	7.4560	52.6342	16.1878	0.7412	6.2015	1.8668
	add-add-max	**7.4568**	**52.6427**	**16.1947**	0.7413	**6.2048**	**1.8671**
Ours	add-add-IRC	*7.5456*	*54.9875*	*17.4877*	0.7495	*6.7154*	*1.8981*
Dataset: NIR							
Group-1	avg-avg-avg	7.1394	43.3521	17.2689	1.0247	6.2557	1.1588
	L1-L1-L1	7.2379	51.7701	19.4998	1.0431	7.2740	1.3302
	max-max-max	7.0997	45.6194	17.0817	**1.1052**	5.9951	0.7921
	add-add-add	7.3349	62.0324	27.6856	1.0332	9.6061	1.5922
	IRC-IRC-IRC	6.8700	39.5068	16.1342	0.0223	5.6700	1.0357
Group-2	avg-add-add	7.1243	42.4781	17.6876	1.0186	6.4665	1.1373
	L1-add-add	7.1960	50.7018	19.6258	1.0309	7.3605	1.2908
	max-add-add	7.1877	47.0221	18.5509	*1.1388*	6.5945	0.8531
	IRC-add-add	6.7393	37.2576	13.6131	0.0294	4.5636	1.0212
Group-3	add-avg-add	7.3022	60.9718	26.9563	1.0258	9.3223	1.5649
	add-L1-add	7.3024	60.9703	26.9508	1.0259	9.3193	1.5651
	add-max-add	7.3041	61.0273	26.9662	1.0263	9.3236	1.5670
	add-IRC-add	6.9583	51.5838	22.6672	0.9350	7.5949	1.2650
Group-4	add-add-avg	7.4244	64.9394	28.4505	1.0556	9.8764	1.6836
	add-add-L1	7.4244	64.9394	28.4505	1.0556	9.8764	1.6836
	add-add-max	**7.4249**	**64.9500**	**28.4522**	1.0557	**9.8770**	**1.6840**
Ours	add-add-IRC	*7.4880*	*66.3561*	*28.7589*	1.0719	*9.9453*	*1.7438*

Considering the existing experimental findings above, almost all evaluation metrics reach best when the low and medium frequency information is based on the addition fusion strategy and the high-frequency feature implements the proposed IRCAFN fusion strategy. Besides, the experimental results also illustrate that the proposed IRCAFN network is more suitable for representative high-frequency details.

#### Comparisons with other state-of-the-art methods

##### Qualitative analysis

**Figure 10:**
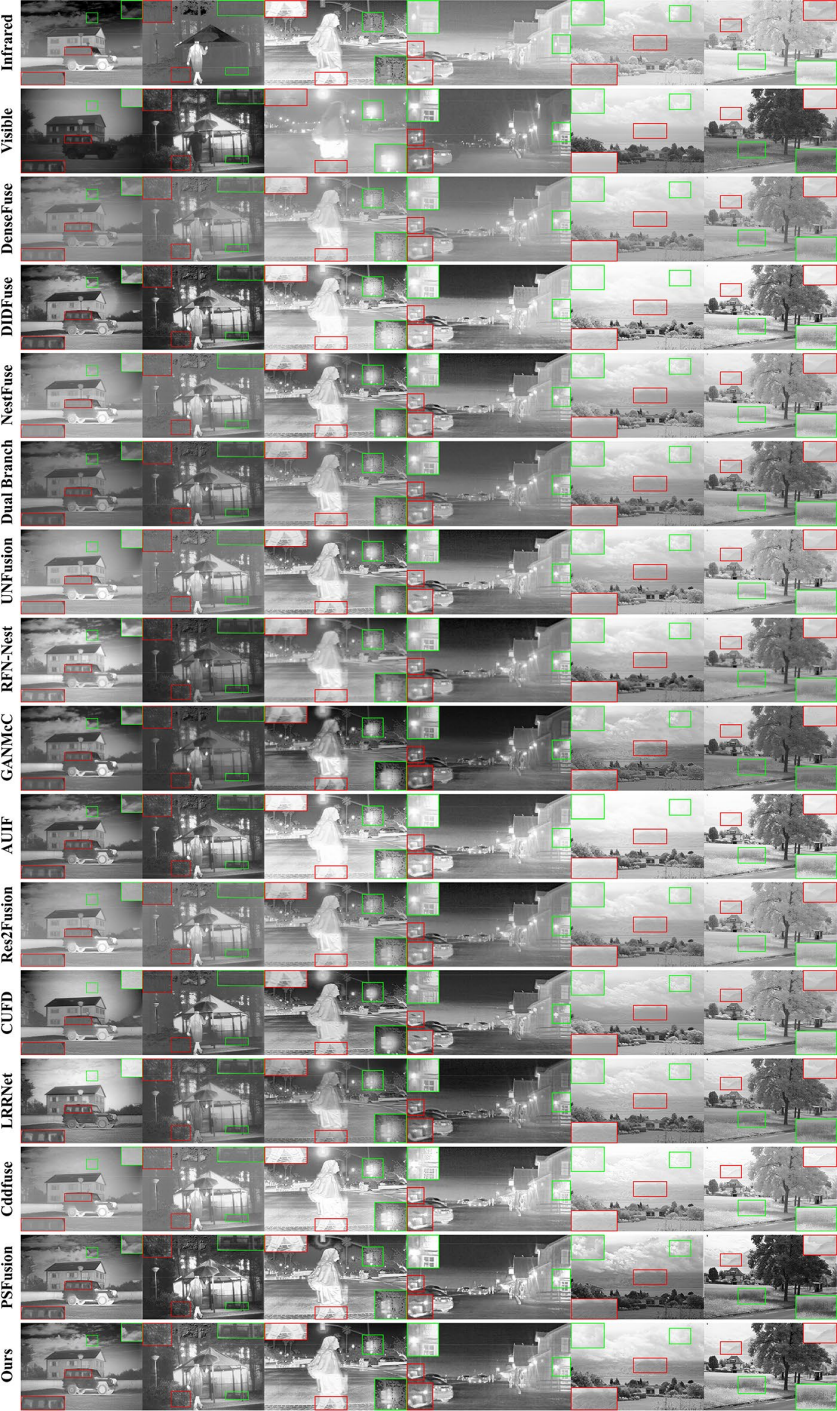
The qualitative comparison results. From top to bottom: infrared images, visible images, results of other methods, and our method.

We compare our method with related methods in the last three years, including DenseFuse^17^, DIDFuse^35^, NestFuse^36^, Dual Branch^37^, UNFusion^38^, RFN-Nest^12^, GANMcC^11^, Res2Fusion^39^, CUFD^40^, LRRNet^41^, Cddfuse^42^, PSFusion^43^, and AUIF^15^. The fusion images are shown in Fig. 10.

To verify the generalization performance of IRCAFN, three prevalent datasets were employed for evaluation. The specific details of the three datasets TNO, FLIR, and NIR are listed in Table 1. It contains day and night scenes, with three types of contents: persons, stuff, and scenery. To fully illustrate the comparison results, red and green boxes are used to subjectively mark and enlarge the selected area.

Firstly, the proposed fusion method maintains the balance of meaningful information between different source images. Fusion images biased toward visible images lead to fusion results containing rich texture information but losing salient infrared information. For example, in the first column of comparison images in Fig. 10, the fusion results of NestFuse, UNFusion, Res2Fusion, CUFD, LRRNet, Cddfuse and PSFusion all lose the cloud detail information in the infrared images. Conversely, other unexpected results, the fusion image contains excessive infrared information with the visible image detail information lost. In the second column of Fig. 10, the results of NestFuse, UNFusion, Res2Fusion, CUFD and Cddfuse do not preserve the information of bench objects in visible images. The comparison of the results can illustrate that the proposed fusion method retains the visible image detail information while highlighting the infrared image saliency target, keeping a well balance between them. In extreme exposure conditions similar with the third column in Fig. 10, the proposed method still permits clearly seeing texture on the figure's pants and the tree branches behind the traffic light.

Furthermore, the proposed fusion method preserves more information. In columns 5 and 6 of Fig. 10, the competitors have lost detailed information of the peaks and grasses to some degree. By contrast, these details are evidently preserved in both our fused images and the PSFusion results. Especially, the comparison image of the detailed information of the distant peaks in column 5 underlines that the proposed method can retain more information about the tiny dim textures.

Finally, our fusion image with high contrast shows clearer details and more consistent with human eye perception. The first column of the DIDFuse appears to have an unnatural brightness distribution, and the RFN-Nest and GANMcC results have a blurred situation. Although the results of DenseFuse, Dual Branch, AUIF and PSFusion exhibit clear details and saliency of the infrared targets, our method demonstrates a higher contrast ratio, which provides clearer details in the images. In addition, the luminance distribution of our results is more uniform with a wider distribution, as visualized in columns 3 and 4 of Fig. 10. So, our visual effect performs better compared to other methods. The distant mountain in column 5 and the tree in column 6 in Fig. 10 both demonstrate that our fusion results are more natural looking and more in line with the human visual perception system.

In general, the fusion results of the proposed method exhibit more detailed information, higher clarity, and are more compatible with the human visual perceptual system.

##### Quantitative analysis

We compared the aforementioned methods quantitatively on three datasets using the above evaluation metrics. The test results are shown in Table 7. In terms of EN, SD, SF, AG, and SCD metrics, the result of our model on three datasets achieves nearly the best or second index. As for the VIF metric, our results achieved a medium standing. Specifically, the larger EN and SCD show that our fusion images retain richer details. It means that the fusion images not only contain rich detail information but also have high contrast and the best visual quality. The best values are obtained in the metrics SF and AG, which represent images containing more detailed edges and higher clarity. Overall, our approach is suitable for IVIF tasks in variable scenes.

**Table 7:** Quantitative results in test datasets. The best and second values are marked in red and black respectively, those are highlighted in bold.

Methods	EN	SD	SF	VIF	AG	SCD
Dataset: TNO image fusion						
DenseFuse^17^	6.3517	24.7822	6.3675	0.6689	2.5151	1.6056
DIDFuse^35^	7.0061	**46.8845**	11.2657	0.8316	4.2942	1.7837
NestFuse^36^	7.0367	41.7506	10.1047	0.9701	3.8921	1.7209
Dual Branch^37^	6.5467	29.5506	6.5968	0.7290	2.6446	1.6788
UNFusion^38^	7.0215	41.9541	10.2676	**0.9990**	3.9066	1.6959
RFN-Nest^12^	6.9907	37.2476	5.8681	0.8234	2.6823	1.7979
GANMcC^11^	6.7474	33.6386	6.1331	0.7125	2.5457	1.7141
AUIF^15^	6.9438	43.4466	11.0526	0.8113	4.1458	**1.8602**
Res2Fusion^39^	7.0035	42.1883	9.9189	0.9986	3.8371	1.6818
CUFD^40^	7.0781	43.2434	10.0307	**0.9997**	4.1002	1.5393
LRRNet^41^	7.0369	41.7683	9.5032	0.8117	3.7888	1.5398
Cddfuse^42^	7.0771	44.8513	12.3941	0.9497	4.7149	1.7746
PSFusion^43^	**7.2555**	**47.8963**	**13.8496**	0.9334	**5.5595**	1.8222
Ours	**7.1258**	45.6097	**13.0833**	0.8363	**5.0197**	**1.8859**
Dataset: FLIR image fusion						
DenseFuse^17^	6.7865	31.0284	8.4573	0.6415	3.3604	1.3938
DIDFuse^35^	7.3442	50.9797	14.2406	0.7145	5.5740	1.7719
NestFuse^36^	7.4228	49.4558	13.7264	**0.8387**	5.1528	1.6944
Dual Branch^37^	6.8904	33.4097	8.8380	0.6836	3.5168	1.4576
UNFusion^38^	7.3687	49.1695	13.6223	**0.8546**	5.0286	1.6325
RFN-Nest^12^	7.2992	44.1390	7.8133	0.6913	3.3895	1.7273
GANMcC^11^	7.2090	42.4833	8.6562	0.6541	3.6745	1.6513
AUIF^15^	7.4333	52.3234	14.9051	0.7384	5.6936	**1.8705**
Res2Fusion^39^	7.3280	46.9290	12.4000	0.7876	4.7219	1.6563
CUFD^40^	7.2986	48.3180	13.9151	0.8140	5.4416	1.4121
LRRNet^41^	7.1328	42.0972	12.4886	0.6738	4.7183	1.5947
Cddfuse^42^	**7.4493**	**55.1451**	**18.6993**	0.7796	6.7884	1.7122
PSFusion^43^	7.4407	50.1545	17.1440	0.7280	**6.9633**	1.6961
Ours	**7.5462**	**54.9989**	**17.7122**	0.7470	**6.7942**	**1.8951**
Dataset: NIR image fusion						
DenseFuse^17^	7.1020	41.0372	14.3573	1.0164	5.2869	1.0889
DIDFuse^35^	7.3566	60.9785	24.6892	1.0114	8.7410	1.5885
NestFuse^36^	7.3650	49.5369	18.4928	**1.1666**	6.7469	1.4093
Dual Branch^37^	7.1557	42.6880	14.2145	1.0424	5.3738	1.1675
UNFusion^38^	7.3506	48.9503	18.6611	**1.1900**	6.7622	1.3779
RFN-Nest^12^	7.3705	50.4780	9.7134	1.0514	4.1925	1.3373
GANMcC^11^	7.1260	43.1612	13.9296	0.8627	5.2544	1.0074
AUIF^15^	7.4042	**63.5707**	25.5714	1.0531	8.9161	**1.6843**
Res2Fusion^39^	7.3327	47.2911	17.6199	1.1407	6.4327	1.3601
CUFD^40^	7.1336	43.9555	16.2832	1.0092	6.4007	1.0360
LRRNet^41^	7.3046	50.6719	17.7585	0.9572	6.6001	1.2116
Cddfuse^42^	7.4223	57.9436	**27.1514**	1.0682	**9.4398**	1.5494
PSFusion^43^	**7.5960**	61.7739	23.2927	1.0743	8.9224	1.5838
Ours	**7.4921**	**66.6089**	**28.7114**	1.0711	**9.9079**	**1.7546**

## Conclusions

To improve the performance of infrared and visible image fusion, this work develops an efficient deep learning fusion model in triple frequency bands. Especially, a medium frequency feature extraction branch is intended to address the complete information preservation, which is based on the DRGN and PSA modules. Considering the specific characteristics of different spectrums, we fuse the features of different frequency bands adaptively, while an interactive residual coordinate attention fusion network is applied for high-frequency feature fusion. More importantly, the contrastive learning is leveraged to train the multiscale decomposition network for enhancing the complementarity of information at different frequency spectra. With the complete multi-scale decomposition and adaptive fusion strategy, more informative contents can be represented in the feature extraction stage and the fusion efficiency can also be enhanced. Both qualitative and quantitative experiments confirm the superiority of our approach over state-of-the-art methods. In the future, we will explore a fully adaptive fusion network based on contrastive learning, which can further boost the effectiveness in different frequency bands for IVIF tasks.

## Data Availability

The datasets generated during and/or analyzed during the current study are available at https://github.com/shazong0526/IRCAFusion. All other used public datasets are available and cited in reference: The TNO database is available on https://figshare.com/articles/dataset/TNO_Image_Fusion_Dataset/1008029, the FLIR database is available on https://www.flir.com/oem/adas/adas-dataset-form, the NIR database is also available on https://ivrlwww.epfl.ch/supplementary_material/cvpr11/index.html.
